# Prognostic significance of neutrophil count on in-hospital mortality in patients with acute type A aortic dissection

**DOI:** 10.3389/fcvm.2023.1095646

**Published:** 2023-03-03

**Authors:** Weiqi Feng, Huili Li, Qiuji Wang, Chenxi Li, Jinlin Wu, Jue Yang, Ruixin Fan

**Affiliations:** ^1^ Department of Cardiovascular Surgery, Guangdong Provincial Cardiovascular Institute, Guangdong Provincial People's Hospital (Guangdong Academy of Medical Sciences), Southern Medical University, Guangzhou, China; ^2^ School of Medicine, South China University of Technology, Guangzhou, China; ^3^ The Second School of Clinical Medicine, Southern Medical University, Guangzhou, China; ^4^ Department of Cardiac Surgery Intensive Care Unit, Guangdong Cardiovascular Institute, Guangdong Provincial People's Hospital (Guangdong Academy of Medical Sciences), Southern Medical University, Guangzhou, China

**Keywords:** inflammation, neutrophil, mortality, type A aortic dissection, NLR

## Abstract

**Backgrounds:**

The goal of this study was to assess the impact of neutrophil count, in patients with acute type A aortic dissection (ATAAD).

**Methods:**

This study retrospectively collected data from patients between September 2017 and June 2021. Youden's index was used to determine the optimal cut-off value for the neutrophil count and patients were divided into two subgroups. A restricted cubic spline (RCS) was used to model the relationship between variables and in-hospital mortality. The least absolute shrinkage and selection operator (LASSO) method and multivariate logistic regression analyses were used to investigate the independent prognostic factors for in-hospital mortality in patients with ATAAD.

**Results:**

A total of 467 patients were enrolled in this study. In-hospital mortality was 7.28%. The group with elevated neutrophil counts had significantly higher mortality than the group with decreased neutrophil counts (10.8% vs. 3.2%, *P* = 0.02). This data shows that elevated neutrophil count was significantly associated with in-hospital mortality (OR 3.07, 95% CI 1.22–7.62, *P* = 0.02).

**Conclusions:**

Neutrophil count is an independent risk factor for in-hospital mortality in patients with ATAAD. It is an effective inflammatory index, which can be individualized for patients.

## Introduction

Aortic dissection, with its high mortality rate, is a rare and lethal disease. The dissection of the aorta allows blood to flow between the layers of the aortic wall, forcing the layers apart (1). Acute type A aortic dissection (ATAAD) accounts for 58%–62% of aortic diseases (2).

Inflammation plays a crucial role in the pathogenesis and progression of cardiovascular disease (3). White blood cell (WBC) count and C-reactive protein (CRP), which are useful and easily available through routine blood tests, are commonly used biomarkers in cardiovascular disease, including ATAAD. Neutrophils make up 50%–70% of circulating WBCs and are an important inflammation factor. With the aggravation of inflammation, the number of neutrophils circulating in the blood rapidly increases. Recent findings indicated that neutrophil accelerated atherosclerosis promoted atherosclerotic plaque instability, and aggravated ischemic stroke (4, 5). The abundance of neutrophils destroys the aneurysmal vessel wall, thereby promoting progressive enlargement and rupture (6).

Despite recent findings, the specific association between neutrophils and the endpoint of patients with aortic dissection remains unclear. This study aimed to assess the impact of neutrophil count in patients with acute type A aortic dissection and focus on whether neutrophil counts have potential value for early detection in patients with ATAAD, and determine if they can aid in identifying patients at increased risk of mortality.

## Methods

### Study setting

A series of consecutive patients enrolled in this retrospective study were admitted to the hospital from September 2017 to June 2021. The inclusion criteria were as follows: 1. All the patients were diagnosed with ATAAD by computer tomography; 2. Patients were ≥ 18 years of age; 3. Routine blood tests were evaluated within 2 h of admission; 4. All patients underwent surgery within 4 days of admission.

This study was approved by the Ethics Committee of Guangdong Provincial People's hospital.

### Data collection

All clinical information, including demographics, admission laboratory results, operative information, and clinical results, were collected from the hospital's medical record system. Admission laboratory tests, including routine blood tests and metabolic profiles, were run within 2 h of arrival to the emergency room. Operative information was collected including cardiopulmonary bypass time (CPB), coronary artery bypass graft (CABG), aortic cross-clamp time (ACC) and whether patients underwent a Bentall procedure. All data from our study were used without *a priori* sample size calculations. And all null value data in the data set were deleted to ensure validity.

### Endpoint

All-cause deaths during hospitalization were defined as the primary endpoint of study. Gastrointestinal bleeding, paraplegia, acute kidney failure, chest reopening, low cardiac output syndrome, cerebrovascular accident, multiple organ dysfunction syndrome (MODS) were also included as the secondary endpoint.

Acute kidney failure was defined as Serum creatinine increased by >3 times the baseline values, GFR decreased by >75%, oliguria: urine output <0.3 ml · kg−1 · h−1 for 24 h, or anuria >12 h or requiring temporary hemodialysis support for resolution (7). Low cardiac output syndrome was defined as large doses of vasoactive drugs with signs of hypoperfusion of tissues, requiring intra-aortic balloon pump insertion or requiring extracorporeal membrane oxygenation support.

### Statistical analysis

Continuous variables are summarized as the mean ± standard deviation and median (inter-quartile range). Categorical variables are summarized as frequency rates and percentages. The differences between the two groups were compared using Student's *t*-test, Mann-Whitney *U* test, and *χ*2 tests.

The Kaplan-Meier method was used to construct the survival curve. Receiver operating characteristic (ROC) curves were constructed, and the area under the curve (AUC) was assessed for analyzing the prognostic value. The continuous variables of laboratory results are determined by Youden's index, which is helpful to determine the optimal cutoff values for dividing subjects into subgroups. A restricted cubic spline (RCS), with three knots at 25th, 50th and 75th, was used to assess the non-linear relationship between neutrophil count and in-hospital mortality.

The least absolute shrinkage and selection operator (LASSO) method was performed. LASSO regression is known to be able to remove unimportant variables *via* the regression coefficients penalizing the size of the parameters (8). Tuning parameter (*λ*) selection in the LASSO model used 10-fold cross-validation *via* minimum criteria. And then, variables with non-zero coefficients in the LASSO-logistic analysis were selected for further stepwise logistic regression analysis. Calibration plot was used to represent perfect prediction that model-predicted probability matches actually observed probability. Clinical utility was estimated by decision curve analysis (DCA). In our study, a two-sided *P*-value of < 0.05 was considered to be significant. Stata software (StataCorp, United States) and R software were used for statistical analysis.

## Results

From September 2017 to June 2021, a total of 477 consecutive patients were included in our study. 10 patients were excluded by the inclusion criteria. The clinical characteristics of all patients are presented in Table 1. 394 (84.37%) patients were male, and the mean age of all patients was 52.0 ± 10.59.

**Table 1:** Baseline characteristics of clinical data in patients.

	Overall (*n* = 467)	Survivor (*n* = 433)	Non-survivor (*n* = 34)	*P*-value
**Demographics**
Age (years)	52.06 ± 10.59	51.74 ± 10.49	56.09 ± 11.12	0.02
Gender/male	394 (84.37%)	367 (84.75%)	27 (79.41%)	0.41
BMI(kg/m^2^)	24.75 ± 3.95	24.73 ± 3.74	24.91 ± 6.12	0.79
Smoking^a^	161 (34.48%)	154 (35.57%)	7 (20.59%)	0.08
**Medical history**
Hypertension	320 (68.52%)	291 (67.21%)	29 (85.3%)	0.03
Diabetes	8 (1.71%)	7 (1.62%)	1 (2.95)	0.57
CAD	46 (9.85%)	40 (9.24%)	6 (17.65%)	0.11
Known history of cardiovascular surgery	38 (8.14%)	36 (8.31%)	2 (5.9%)	0.62
MFS	24 (5.14%)	24 (5.54%)	0 (0.0%)	0.16
Aortic regurgitation				0.03
Non/mild		293 (69.1%)	18 (52.9%)	
Moderate		74 (17.5%)	13 (38.2%)	
Severe		57 (13.4%)	3 (8.8%)	
**Laboratory results**
White blood cell count ( ×10^9^)	12.80 ± 3.79	12.74 ± 3.86	13.59 ± 2.75	0.21
Neutrophil ( ×10^9^)	10.23 ± 3.65	10.12 ± 3.67	11.70 ± 2.98	0.01
Lymphocyte ( ×10^9^)	1.07 (0.83–1.44)	1.08 (0.85–1.44)	1.02 (0.69–1.47)	0.30
Platelets ( ×10^9^)	184 (152–228)	185 (152–228.5)	164 (146.5–211)	0.07
NLR	9.22 (6.13–13.45)	8.86 (6.03–13.18)	11.42 (8.00–16.80)	<0.01
Serum creatinine (g/dl)	90.00 (71.10–115.33)	89.39 (70.83–111.89)	103.12 (82.73–137.64)	0.73
AST	24.0 (19.0–34.0)	24.0 (18.0–34.0)	28.5 (21.8–36.8)	0.67
ALT	21.0 (14.8–32.0)	21.0 (14.0–32.0)	24.0 (17.5–30.0)	0.64
**Operative information**
CPB time (minute)	240.5 (210.0–279.0)	236.5 (208.0–274.0)	298.5 (259.5–365.3)	<0.01
CABG	31 (6.64%)	18 (4.16%)	13 (38.23%)	<0.01
ACC time (minute)	134.3 ± 41.1	131.3 ± 37.8	172.0 ± 59.5	<0.01
Bentall	107 (22.91%)	100 (23.09%)	7 (20.59%)	0.74
Total arch replacement	449 (96.15%)	416 (96.07%)	33 (97.06%)	0.77

^a^ Smoking is defined as current smoking (smoking more than 100 cigarettes and having smoked in the last 1 month) and ex-smoking.

BMI: body mass index; CAD: coronary arterial disease; MFS: Marfan syndrome; NLR: neutrophil to lymphocyte ratio; AST: aspartate aminotransferase; ALT: Alanine aminotransferase; CPB: cardiopulmonary bypass; ACC: aortic cross-clamp time; CABG: coronary artery bypass graft.

### Clinical characteristics of patients

Patients were divided into a survivor group (*n* = 433) and a non-survivor group (*n* = 34). The age in the survivor group was younger than in the non-survivor group (51.74 ± 10.49 vs. 56.09 ± 11.12, *P* = 0.02). Hypertension (*P* = 0.03), and CABG procedures (*P* < 0.01) were more commonly found in the non-survivor group. Patients had longer CPB and ACC time (*P* < 0.01), higher neutrophil to lymphocyte ratio (NLR; *P* < 0.01), and higher neutrophil counts (10.12 ± 3.67 vs. 11.70 ± 2.98, *P* = 0.01) in the non-survivor group. There were no significant differences between the two groups in other measured variables (Table 1).

### Neutrophil count and endpoints

Youden's index was used to determine the optimal cut-off value for the neutrophil count, and then, patients were divided into two subgroups: the decreased neutrophil group (≤9.59 × 10^9^/L), and the elevated neutrophil group (>9.59 × 10^9^/L). As shown in Table 2, the elevated neutrophil group (>9.59 × 10^9^/L) patients were more likely to present with death (*P* < 0.01), gastrointestinal bleeding (*P* = 0.04) and low cardiac output syndrome (*P* = 0.02) than the decreased neutrophil group patients. There were no significant differences between two groups in other endpoints.

**Table 2:** The endpoints of the different neutrophil groups.

Endpoints	Neutrophil ≤ 9.59 × 109/L (*n* = 217)	Neutrophil > 9.59 × 109/L (*n* = 250)	*P*-value
Mortality	7 (3.2%)	27 (10.8%)	<0.01
Gastrointestinal bleeding	2 (0.9%)	10 (4.0%)	0.04
Paraplegia	8 (3.7%)	12 (4.8%)	0.55
Acute kidney failure	34 (15.7%)	56 (22.4%)	0.07
Reopen the chest	2 (0.9%)	5 (2.0%)	0.34
Low cardiac output syndrome	6 (2.8%)	19 (7.6%)	0.02
Cerebrovascular accident	8 (3.7%)	18 (7.2%)	0.10
MODS	3 (1.4%)	9 (3.6%)	0.13

MODS: multiple organ dysfunction syndrome.

**Figure 1:**
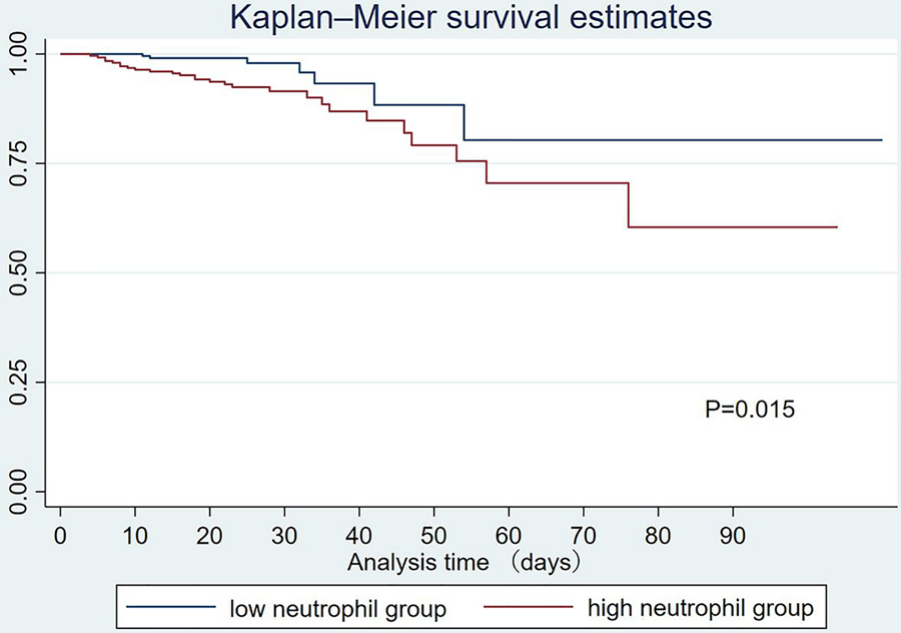
Kaplan-Meier analysis for in-hospital mortality by different neutrophil counts. The survival difference was significant (*P* = 0.015).

**Figure 2:**
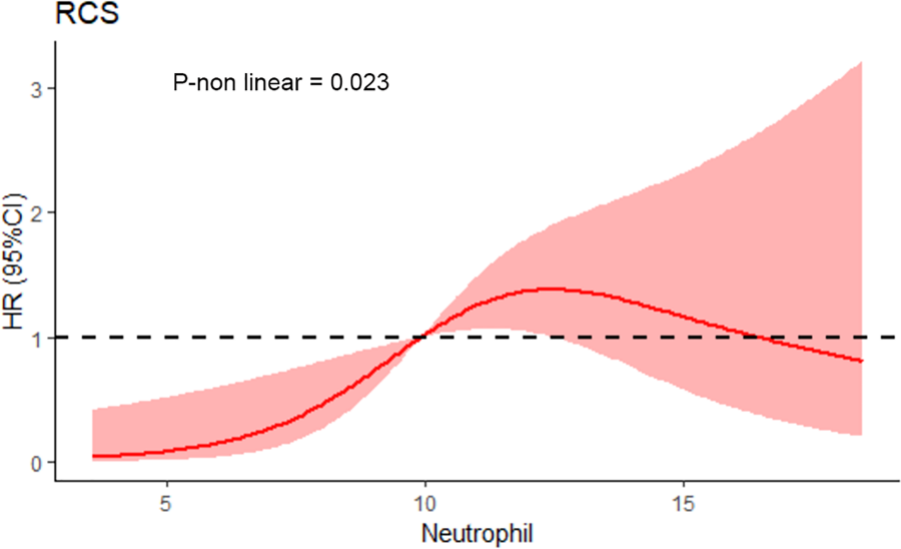
Restricted cubic splines of neutrophil count and in-hospital mortality. *(P non-linear = 0.023)*.

Total in-hospital mortality was 7.28%. The in-hospital mortality rate was 10.8% in the elevated neutrophil group and 3.2% in the decreased neutrophil group. Figure 1 shows that the cumulative probability of the overall survival between the two groups was statistically significant, with patients in the elevated neutrophil group having a higher rate of in-hospital mortality. A restricted cubic spline was used to model the non-linear relationship between the neutrophil count and in-hospital mortality. As shown in Figure 2, the neutrophil count was the risk factor for in-hospital mortality when 9.9–16.4 × 10^9^/L.

### Logistic regression methods

**Figure 3:**
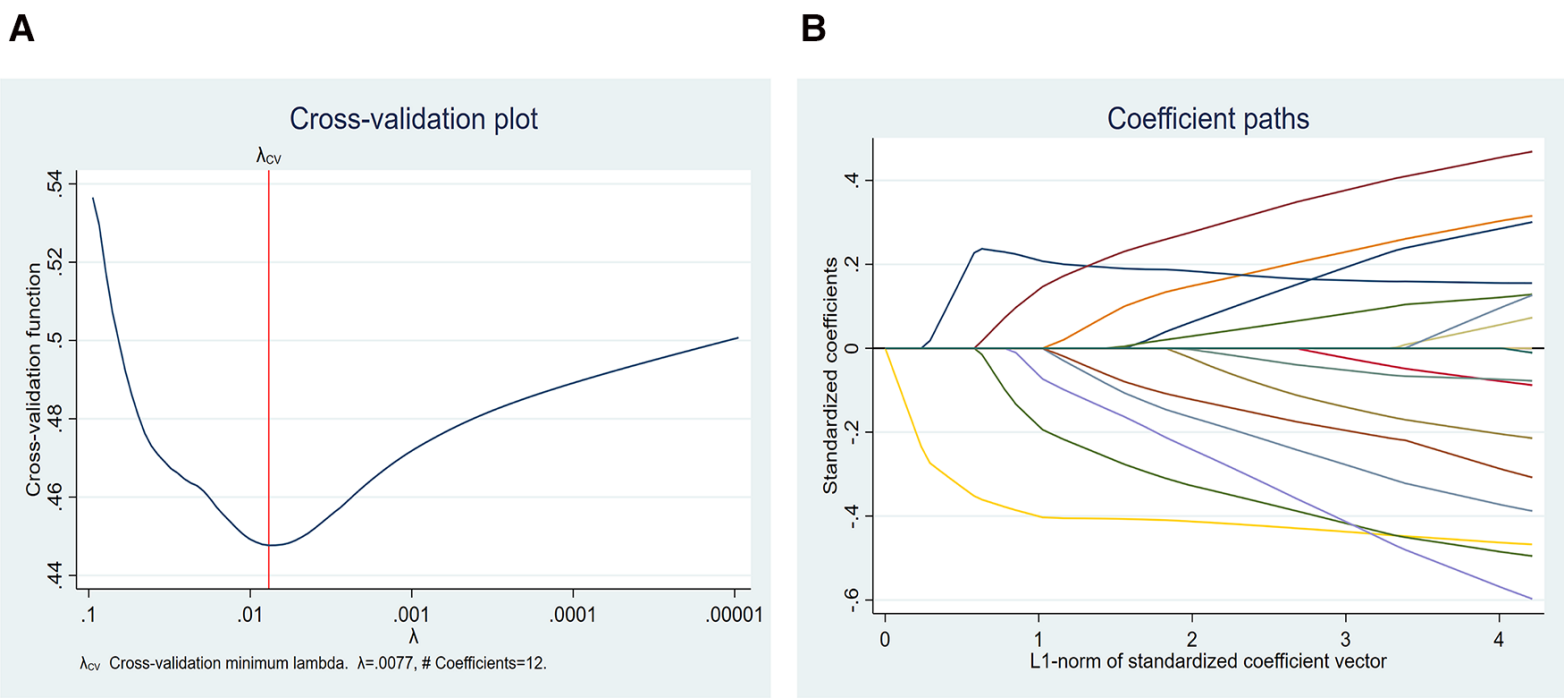
LASSO regression showed log (*λ*) = 0.0077 when the model error was minimized, and 12 variables were selected for further logistic regression analysis.

LASSO analyses were performed to evaluate the risk factor of in-hospital mortality in patients with ATAAD using 10-fold cross-validation (Table 3). LASSO regression showed log (*λ*) = 0.0077 when the error of the model is minimized, and 12 variables (gender, age, smoking, hypertension, diabetes, CABG, CPB, ACC, neutrophil count, NLR, creatinine) were selected with non-zero coefficients for further analyses (Figure 3).

**Table 3:** Risk factors selected by the LASSO-logistic regression model.

Variables	Coefficient
Gender/male	0.127
Age	0.055
Smoke/no	0.188
Hypertension/no	−0.219
Diabetes/no	−0.090
CABG/no	−0.424
CPB	0.170
ACC	0.328
Neutrophil ≤ 9.59	−0.370
NLR ≤9.18	−0.029
Creatinine ≤92.24	−0.324

CABG: coronary artery bypass graft; CPB: cardiopulmonary bypass; ACC: aortic cross-clamp time; NLR: neutrophil to lymphocyte ratio; AST: aspartate aminotransferase.

Multivariate logistic regression analysis then incorporated factors that were selected in the LASSO analyses (Table 4). In the model, elevated neutrophil counts (OR 3.07, 95% CI 1.22–7.62, *P* = 0.02), CABG (OR 9.54, 95% CI 3.47–26.20, *P* < 0.01), elevated serum creatinine (OR3.06, 1.23–7.62, *P* = 0.02) and ACC time (OR 1.01, 95% CI 1.00–1.02, *P* < 0.01) were significant independent risk factors for in-hospital mortality in patients with ATAAD.

**Table 4:** Multivariate stepwise logistic regression analysis of risk factors selected by the LASSO-logistic model.

Variables	Odds ratio	95% CI	*P*-value
Gender/male	0.33	0.11–0.95	0.04
Neutrophil > 9.59	3.07	1.22–7.75	0.02
Creatinine > 92.24	3.06	1.23–7.62	0.02
CABG/with	9.54	3.47–26.20	<0.01
ACC	1.01	1.00–1.02	<0.01

CABG: coronary artery bypass graft; ACC: aortic cross-clamp time.

**Figure 4:**
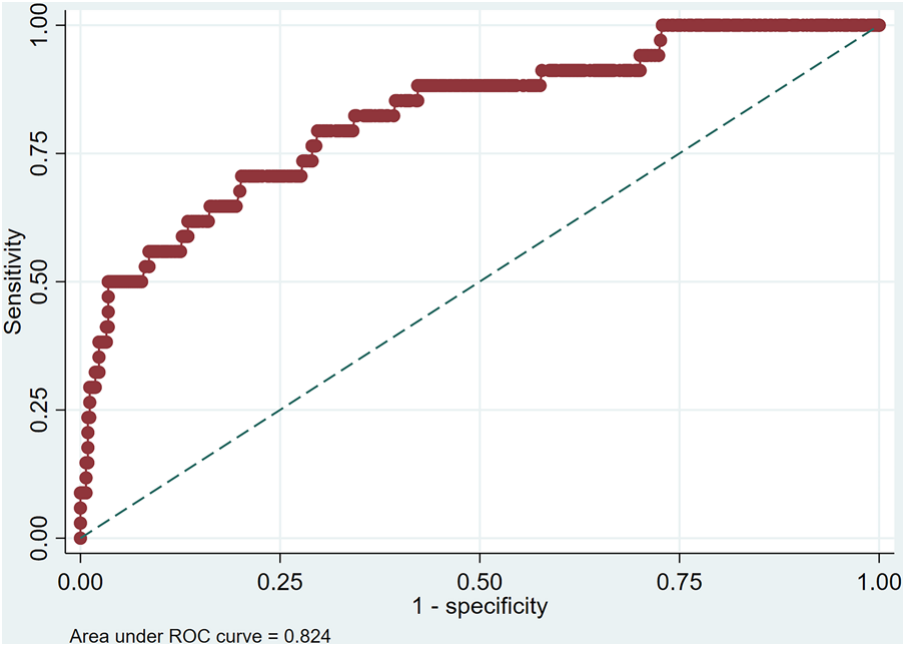
Receiver operating characteristic (ROC) curves of the multivariate logistic regression.

**Figure 5:**
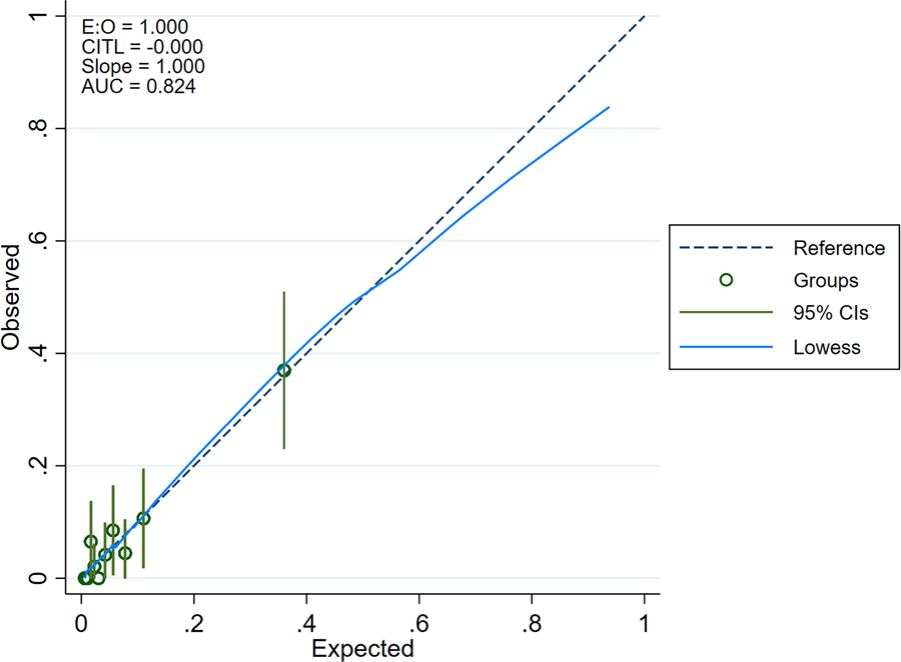
Calibration plot of the model that represents perfect prediction that model-predicted probability matches actually observed probability.

**Figure 6:**
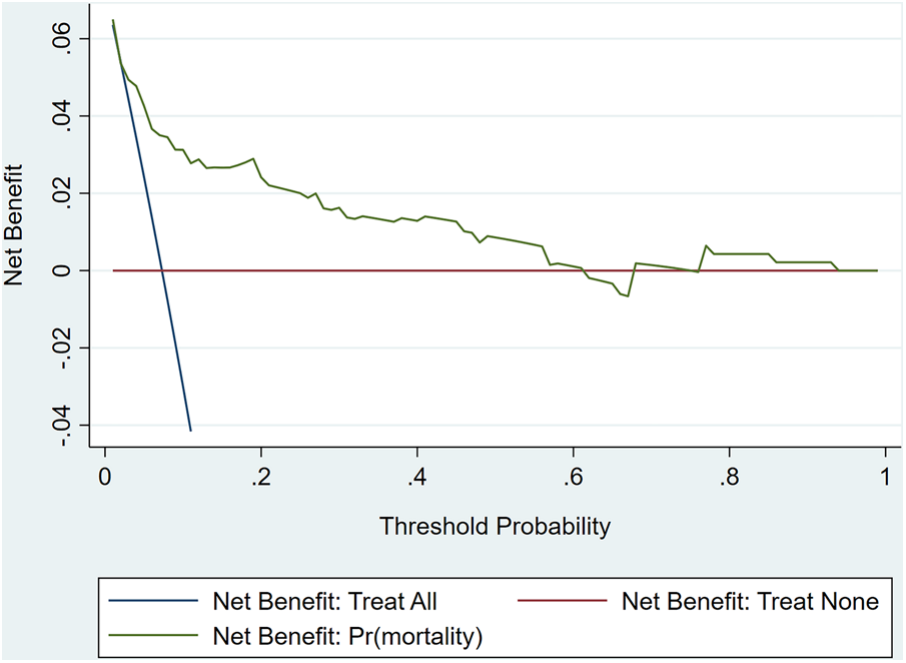
Decision curve analysis of the model.

The ROC curve analysis of model was shown in Figure 4, and the AUC is 0.824. Calibration plot of the model that represents perfect prediction that model-predicted probability matches actually observed probability was shown in Figure 5. As shown in Figure 6, the DCA for mortality in the nomogram of the model was built. When the threshold probability of occurrence of in-hospital mortality was around 0.02 to 0.62, the net benefit level of nomogram is obviously higher than that of “treat all” and “treat none”, which indicates that the nomogram has good clinical applicability.

### Discussion

Aortic dissection is a very serious health condition, and timely diagnosis is crucial to saving lives. Neutrophil count is a classical biomarker of inflammation and a central player during acute inflammatory responses. Our findings helped to individualize the patients and may help to reduce in-hospital mortality of patients in the future.

Mechanical injury induced the expression of neutrophil chemoattractant (9). The release of inflammatory factors enhances the inflammatory reaction of the adventitia following the onset of ATAAD, leading to further dissection of the aorta and rupture of the dissection (10, 11). In-hospital mortality was 7.28% in our study. A former study reported that the mortality rates among ATAAD patients who do not receive surgical treatment can reach 30% within 48 h, proving that surgery is still a crucial method to treat patients. When the patient was diagnosed with acute type A aortic dissection, we will arrange surgery for the patient as soon as possible to reduce the impact of the surgery intervention time on the patient. A previous study has proven that elevated levels of inflammatory markers are predictive of cardiovascular events (12). It may be due to injury that a large number of inflammatory factors are released, causing the increase of central granulocytes. The main finding of this study was that elevated neutrophil count was an independent risk factor for in-hospital mortality (*P* = 0.02). Our study also found that gastrointestinal bleeding and low cardiac output syndrome were more common in patients with the elevated neutrophil count. Therefore, neutrophil plays a key role in the development of AD. The increase in the number of neutrophils should be a warning. When the patient's neutrophil number increases fast, the health system will issue an alarm indicating a critical value, which will help to improve clinical vigilance.

WBC count is the most commonly used blood parameter for many medical conditions. Ma et al. found that WBC > 11 × 10^9^/L was an independent risk factor for mortality in ATAAD patients (OR = 3.10, *P* < 0.01) (13). Our research showed that there was no significant difference in the WBC counts of patients between the survivor and non-survivor groups (*P* = 0.21). This study found that neutrophil count was greatly different in the survivor and non-survivor groups. As a cost-effective and convenient measurement, neutrophil count is a more representative and sensitive inflammation biomarker than NLR and WBC count in this study. NLR and PLR are emerging biomarkers with the combination of hemostatic and inflammatory pathways, which are regarded as potential biomarkers to predict outcomes of cardiac patients. Previous studies have reported the association between NLR, PLR, and in-hospital mortality in patients with cardiovascular diseases, including heart failure, acute coronary syndrome, etc (14–16). A study by Karakoyun et al. showed that elevated NLR may predict in-hospital mortality in patients with ATAAD (15). However, we did not find it to be a risk factor for in-hospital mortality after building a multivariate logistic regression model. Therefore, there was not sufficient evidence to indicate a better predictive value of NLR over neutrophil count in this study. Whether NLR can be a predictive biomarker need further investigation.

RCS found that when neutrophil <9.9 or >16.4 × 10^9^/L, it was not the risk factor for in-hospital mortality. The whole curve is approximately C-shaped. Some patients not only suffered from extensive aortic injuries, but also may suffer from stress reactions, which resulted in an abnormal increase of neutrophils that can't predict the patient's physical condition well.

The multivariate regression model showed that ACC time (OR 1.01, 95% CI 1.00–1.02, *P* < 0.01) and CABG surgery (OR 9.54, 95% CI 3.47–26.20, *P* < 0.01) were significantly related to in-hospital mortality. Although CPB was selected in the LASSO analyses, it was not selected in multivariate logistic regression analyses, and it was still of great significance to prognosis. Some previous studies have illustrated that longer CPB time was associated with a greater possibility of suffering from adverse events in ATAAD patients (OR = 1.01) (17). Shorten CPB and ACC time, reduce organ ischemia-reperfusion injury, and improve the prognosis of the aortic disease. CABG surgery is a high-risk procedure, and postoperative complications can result in significant morbidity and mortality (18). Patients with overlapping surgery facing higher risks may have poorer prognoses.

This research had some limitations. First, it's a single-center retrospective study which undermines statistical power. Therefore, external validation of patients from other hospitals is needed. Secondly, detailed information about timing of intervention is not available in our study, which may have an effect on the results. Finally, the changes in neutrophil counts in acute and chronic patients need further study. Further research is needed to apply this prediction model in clinical practice.

## Conclusion

Based on our research, neutrophil count is independently associated with in-hospital mortality in patients with ATAAD, which is an effective indicator of inflammation and has the potential to help surgeons make decisions regarding treatment strategy going forward.

## Data Availability

The original contributions presented in the study are included in the article/Supplementary Material, further inquiries can be directed to the corresponding author.
